# Cured by the Kick of a Horse

**Published:** 1853-01

**Authors:** G. S. Crawford

**Affiliations:** Joliet, Ill.


ARTICLE IV.—*Cured by the Kick of a Horse*, by G. S. CRAWFORD, M. D. Joliet, Ill.

SOME eight years ago, I was called on by a farmer residing in this neighborhood, a healthy muscular man of active habits, aged about thirty-five, for advice respecting varicose veins in the left lower extremity. I found the veins considerably distended, causing no pain nor uneasiness. He said he had a constant dread of their giving way. I advised the use of a bandage or laced stocking, which he did not use. Time passed, and although intimate with and frequently meeting him, I heard no more from him on the subject, until a few days past. He told me that about four months past he had had a kick from a horse on the inner side of the left thigh, a few inches above the knee. This was followed by slight swelling and pain. Soon afterwards he perceived that the veins, formerly troublesome, were no longer so, and in about ten days they had disappeared. Such is his story of Horse Surgery. At present the limb offers no appearance of disease.

